# Phytochemical, Morphological and Genetic Characterisation of *Anacyclus pyrethrum* var. *depressus* (Ball.) Maire and *Anacyclus pyrethrum* var. *pyrethrum* (L.) Link

**DOI:** 10.3390/molecules28145378

**Published:** 2023-07-13

**Authors:** Fatima Zahra Jawhari, Hamada Imtara, Nabil Radouane, Abdelfattah El Moussaoui, Imane Es-safi, Amal Amaghnouje, Mashail N. AlZain, Omer Noman, Mohammad Khalid Parvez, Dalila Bousta, Amina Bari

**Affiliations:** 1 Laboratory of Biotechnology, Environment, Agri-Food and Health (LBEAS), Faculty of Sciences, Sidi Mohamed Ben Abdellah (USMBA) University, P.O. Box 2202, Fez 30000, Morocco; abdelfattah.moussaoui@usmba.ac.ma (A.E.M.); imane.safi@usmba.ac.ma (I.E.-s.); ama.amaghnouje@usmba.ac.ma (A.A.); dalila.ousta@usmba.ac.ma (D.B.); amina.ari@usmba.ac.ma (A.B.); 2 Faculty of Sciences, Arab American University Palestine, Jenin P.O. Box 240, Palestine; 3 African Genome Center, Mohammed VI Polytechnic University (UM6P), Lot 660, Hay Moulay Rachid, Ben Guerir 43150, Morocco; nabil.radouane@usmba.ac.ma; 4 Department of Biology, College of Sciences, Princess Nourah bint Abdulrahman University, P.O. Box 84428, Riyadh 11761, Saudi Arabia; mnalzain@pnu.edu.sa; 5 Department of Pharmaceutical Biology, Institute of Pharmacy, University of Greifswald, 17489 Greifswald, Germany; omar.noman@uni-greifswald.de; 6 Department of Pharmacognosy, College of Pharmacy, King Saud University, P.O. Box 2457, Riyadh 11451, Saudi Arabia; mohkhalid@ksu.edu.sa

**Keywords:** *Anacyclus pyrethrum* var. *pyrethrum* (L.) Link, *Anacyclus pyrethrum* var. *depressus* (Ball.) Maire, morphological characterisation, physicochemical characterisation, genetic characterisation

## Abstract

The present study is based on a multidisciplinary approach carried out for the first time on *Anacyclus pyrethrum* var. *pyrethrum* and *Anacyclus pyrethrum* var. *depressus*, two varieties from the endemic and endangered medicinal species listed in the IUCN red list, *Anacyclus pyrethrum* (L.) Link. Therefore, morphological, phytochemical, and genetic characterisations were carried out in the present work. Morphological characterisation was established based on 23 qualitative and quantitative characters describing the vegetative and floral parts. The phytochemical compounds were determined by UHPLC. Genetic characterisation of extracted DNA was subjected to PCR using two sets of universal primers, rbcL a-f/rbcL a-R and rpocL1-2/rpocL1-4, followed by sequencing analysis using the Sanger method. The results revealed a significant difference between the two varieties studied. Furthermore, phytochemical analysis of the studied extracts revealed a quantitative and qualitative variation in the chemical profile, as well as the presence of interesting compounds, including new compounds that have never been reported in *A. pyrethrum*. The phylogenetic analysis of the DNA sequences indicated a similarity percentage of 91%. Based on the morphological characterisation and congruence with the phytochemical characterisation and molecular data, we can confirm that *A. pyrethrum* var. *pyrethrum* and *A. pyrethrum* var. *depressus* represent two different taxa.

## 1. Introduction

The expression of intraspecific variability is not only morphological; it can also concern biochemical and genetic traits [1]. The main works of systematic botany of species are based on a set of characters expressed at the morphological (flower, leaf, fruit, seed, cotyledons, pollen grains, and nodules), phytochemical (characterisation at the level of secondary and primary metabolites), and genetic (characterisation based on RNA and DNA nucleic acids) levels [2,3,4,5,6].

Morphological characterisation of a plant is a critical and important trait as it characterises growth, developmental profile and plays a role in formulating strategies for conservation [7,8,9]. For the distinction between taxa, it is important to take into account the different levels and degrees of variation, both intraspecific and interspecific [1].

Phytochemical variability is the result of the expression of the genetic heritage of the species [10], but it can also be linked to different external factors to which the plant is subjected throughout its development, such as environmental conditions (temperature, light, rainfall, and edaphic conditions) [11,12], vegetative stage [10], plant organ [13], season, microorganism attacks, and competition [7]. All these factors make it difficult to standardise phytochemical compositions from two varieties of the same plant species [14]. 

Interest in molecular characterisation is emerging in taxonomy, plant breeding, variety protection, and genetic resource management. The genetic diversity is the extent of genetic variability measured at the scale of an individual, population, metapopulation, species, or group of species [15,16,17]. In addition, the diversity and genetic structure of plant species vary according to their reproductive system, life cycle, geographical distribution, taxonomic status [18], and the size of the seed supply in the soil [19,20]. However, there is not only abundant species diversity, but also significant genetic variability (variants between individuals within a species). Through genetic variability and within the limits of the species, individuals differ from each other in one or more traits. To examine the relationships of similarities and differences between individuals of the same species (intraspecific variation) or of different species (interspecific variation), variation at the genome level offers many advantages over morphological data because variation detected by molecular analysis of DNA can be quantified and is not subject to environmental effects [2,21,22]. Molecular biology has identified specific genetic markers that can distinguish the difference between varieties and species [23,24,25].

Medicinal species, such as *Anacyclus pyrethrum* (L.) Link, were identified in 1979 with two varieties: *Anacyclus pyrethrum* var. *pyrethrum* (L.) Link (*A.P* var. *pyrethrum)* and *Anacyclus pyrethrum* var. *depressus* (Ball.) Maire (*A.P* var. *depressus)* [26,27,28]. Is an endangered medicinal species endemic to Morocco, Spain, and Algeria. It is a gynomonic species with a mixed autogamy–allogamy reproductive cycle with a strong allogamic predominance [29]. The species is well known for its many medicinal properties. In traditional medicine, the roots of *A. pyrethrum* are recommended for treating salivary secretion, even paralysis of the tongue and limbs, toothache, angina, female infertility, lethargy, and digestive problems. They are used in the form of cream-based animal fats to treat gout and sciatica and keep illness away [30]. Other pharmacological and biological properties of *A. pyrethrum* have been reported in the literature, such as aphrodisiac [31,32,33,34,35,36], androgenic and fertilising [35,37,38,39], anti-amnesiac [40], immunostimulant [37,41], muscle relaxers [42], insecticide [43,44,45], antimicrobial [46,47], antibacterial [48,49], antifungal [50], sialagogue [51,52,53], antidepressant [54], anticonvulsant [31,40,55,56], analgesic [57,58], anti-inflammatory [58,59,60], antioxidant [36,40,49,61], antidiabetic [62,63,64,65], anti-cancer [66], and memory enhancers [67,68]. These properties are the result of a wide variety of phytochemical compounds, of which a hundred different compounds have been described to date, such as phenolic compounds, flavonoids, alkaloids, tannins, resinous substances, gum, traces of volatile oil, and also trace elements (Bi, Cu, Fe, K, Mg, Mn, Na, P, Se, and Zn) [31,32,37,42,49,58,69,70,71,72,73,74,75,76,77].

To the best of our knowledge, no previous research has investigated the morphological, phytochemical, or genetic characterisation of the two varieties of *A. pyrethrum*. Thus, in the present work, we opted for a multidisciplinary approach carried out for the first time on the two varieties *A.P* var. *pyrethrum* and *A.P* var. *depressus* and their different parts by morphological, phytochemical, and genetic characterisation for the benefit of their differentiation, valorisation, and conservation.

## 2. Results and Discussion

### 2.1. Morphological Characterisation

#### 2.1.1. Descriptive Analysis of Qualitative Characteristics

For the qualitative characterisation, seven qualitative morphological descriptors were considered. The variability for each of the qualitative descriptors was analysed separately. Correspondence factor analysis (CFA) allowed us to determine the correspondence between several independent characteristics by considering the five qualitative descriptors of high variability for the characteristics of leaf base appearance, corolla back colour, shape and colour of the seed, and root colour. Table 1 shows the qualitative variability assessed for each of the two varieties.

Table 1 shows the two varieties are distinguishable from one another by the colour of the back of the petals, which are red in the *pyrethrum* variety and violet in the *depressus* variety. The seeds are also different between the two varieties, with the *depressus* variety having thick, light-coloured wings and the *pyrethrum* variety having thin, dark-coloured wings. The *depressus* variety’s roots are light brown, whereas the *pyrethrum* variety’s roots are dark brown. At the level of the leaves, the *pyrethrum* variation has an evergreen base, whereas the *depressus* variety does not.

**Figure 1:**
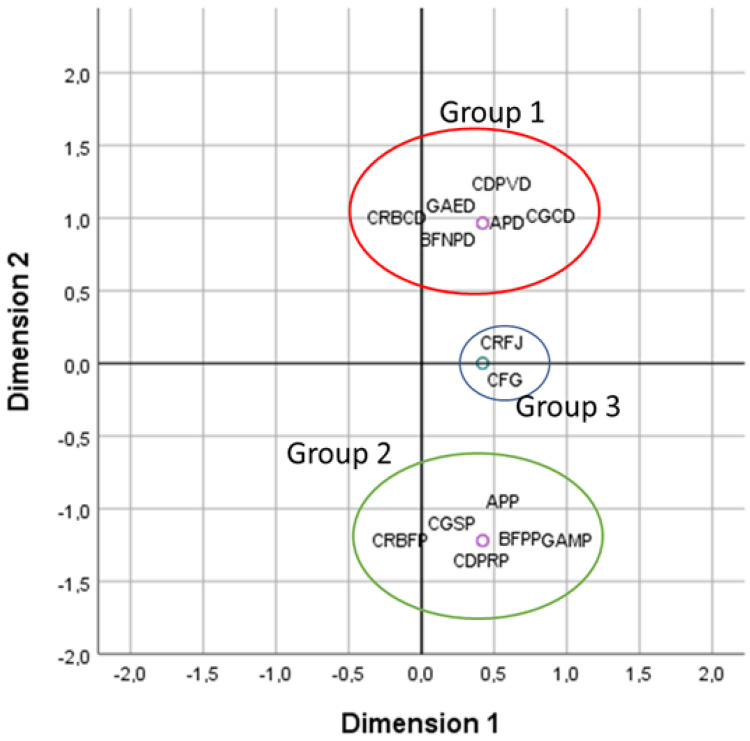
Projection of the qualitative characteristics of the two varieties studied on the plane formed by the two axes of the CFA. APP: *A.P* var. *pyrethrum*; APD: *A.P* var. *depressus*; CRBFP: dark brown root colour of *A.P* var. *pyrethrum*; CGSP: dark seed colour of *A.P* var. *pyrethrum*; CDPRP: red petal back colour of *A.P* var. *pyrethrum*; BFPP: evergreen leaf base of *A.P* var. *pyrethrum*; GAMP: thin wings of *A.P* var. *pyrethrum*; CFG: glaucous leaves; CRFJ: yellow floral ray; CDPVD: violet petal back colour of *A.P* var. *depressus*; CGCD: clear seed colour of *A.P* var. *depressus*; BFNPD: not evergreen base of *A.P* var. *depressus*; GAED: thick wings of *A.P* var. *depressus*; CRBCD: light brown root colour of *A.P* var. *depressus*.

In order to determine which qualitative characteristics are the most discriminating and suitable for morphological characterisation and classification of varieties, a correspondence factor analysis (CFA) was carried out on seven qualitative characteristics. The projection of the qualitative characteristics onto the plane formed by the two axes of the CFA shows variability between the two varieties evaluated. This is shown by the dispersion of the scatter plot representing the different characteristics (Figure 1) in the form of three groups.

Figure 1 shows the appearance of three groups: the first group consists of the qualitative characters that relate to the variety *A.P* var. *depressus*; the second group contains the characters that belong to the variety *A.P* var. *pyrethrum*; and the third group presents the common characters between the two varieties, namely leaf colour and floral ray.

This analysis demonstrates that there are differences between the two varieties in terms of the considered qualitative characteristics.

#### 2.1.2. Descriptive Analysis of Quantitative Morphological Traits Studied

- Diversity of quantitative morphological characteristics

The mean, minimum, and maximum values of the quantitative variables are shown in Table 2 and Table 3.

Significant differences are observed between the minima and maxima for characters such as number of branches (FNR), number of tubular flowers per capitula (NFT), length of root (LOR), number of capitula per individual (NC), average number of seeds per capitula (NG), and weight of 100 grains (PG). On average, the length of the root varies from a mean value of 6.637 ± 1.110 cm for the variety *A.P* var. *depressus* to a mean value of 13.979 ± 2.188 cm for the variety *A.P* var. *pyrethrum*. The average number of seeds per capitula ranged from 116.98 ± 21.75 for *A.P* var. *pyrethrum* to 81.73 ± 22.45 for *A.P* var. *depressus*. The weight of one hundred seeds varied from 0.05 g for *A.P* var. *depressus* to 0.13 g for *A.P* var. *pyrethrum*. The average number of capitula per individual varies with an average of 46.33 ± 10.094 for the variety *A.P* var. *pyrethrum* and 89.32 ± 29.80 for the variety *A.P* var. *depressus*, while the number of tubular flowers per capitula also varies from one variety to another; in fact, the variety *A.P* var. *pyrethrum* has more tubular flowers (117.36 ± 27.509) than the variety *A.P* var. *depressus* (78.05 ± 25.920). Regarding the size of the tubular flowers, there was no significant difference between the two varieties. The variability in the number of flowers per capitula could be explained by the size of the capitula. The most obvious difference is in the size of the roots of *A.P* var. *pyrethrum*, which has long roots, while those of *A.P* var. *depressus* are shorter. The differences observed between the minima and maxima for the studied characters can be explained by the age of the individuals.

- Descriptive statistics for quantitative characteristics

The coefficient of variation (CV) for the 16 quantitative morphological characteristics recorded on the two varieties is presented in Table 4.

The three least variable characters between individuals of the variety *A.P* var. *depressus* are the number of ligulate flowers per capitula (CV = 7.43%), width of tubular flowers (CV = 5.94%), and width of seeds (CV = 8.54%). While the most variable characters are the number of capitula per individual (CV = 33.36%), the number of tubular flowers and seeds per capitula (CV = 33.21%; CV = 25.59%, respectively), and the number of branches per individual (CV = 24.86%), for the variety *A.P* var. *pyrethrum,* the characters that vary the least between individuals are the length and width of the seeds (CV = 3.79%; CV = 5.52%, respectively) and the length of the tubular and ligulate flowers (CV = 7.93%; CV = 6.67%, respectively). The most variable characters were the number of branches per individual (CV = 31.62%) and the width of ligulate flowers (CV = 24.96%). In addition, most of the quantitative characters studied show greater variability between the two varieties than within the variety.

In general, significant variations between the two varieties were found (Table 4); the analysis of variance revealed highly significant differences (*p* < 0.001). The results indicate that among the 16 traits examined, the 8 most discriminating traits were the number of seeds per capitula, the weight of 100 seeds, the number of tubular flowers per capitula, the length and width of Ligulate flowers, the number of capitula per individual, the number of branches, and the length of the roots.

- Correlation between quantitative morphological characteristics

The correlation coefficient quantifies the degree of association or variation between the two descriptors. The sign of the coefficient indicates the type of association: positive (+) if the relationship is direct and negative (−) If the relationship is inverse. If the coefficient approaches 1, the two descriptors are closely correlated [19]. Table S1 shows the correlation coefficients obtained between the 16 quantitative traits measured. These analyses show the presence of significant positive and negative correlations between all the characteristics studied. In particular, they show the presence of highly significant positive correlations between characteristics describing the same variety and a highly significant negative correlation between characteristics concerning the variety *A.P* var. *pyrethrum* and those concerning the variety *A.P* var. *depressus*. As the significant correlations generally concern different parts of the two varieties, no characteristics could be eliminated as a result of this analysis.

The highest positive correlation coefficient (r = 0.99) was observed between the length and width of capitula (LOCP, LACP, LOCD, and LACD). The highest positive correlation coefficient (r = 0.99) was observed between the length and width of flower capitula (LOCP, LACP, LOCD, and LACD) and the number, length, and width of ligulate flowers (NFLP, LOFLP, and LARFLP) (Table S1).

Furthermore, the results of Bartlett’s sphericity test and the overall KMO index for the matrix are significant, which confirms that the data matrix can be subjected to exploratory factor analysis.

**Figure 2:**
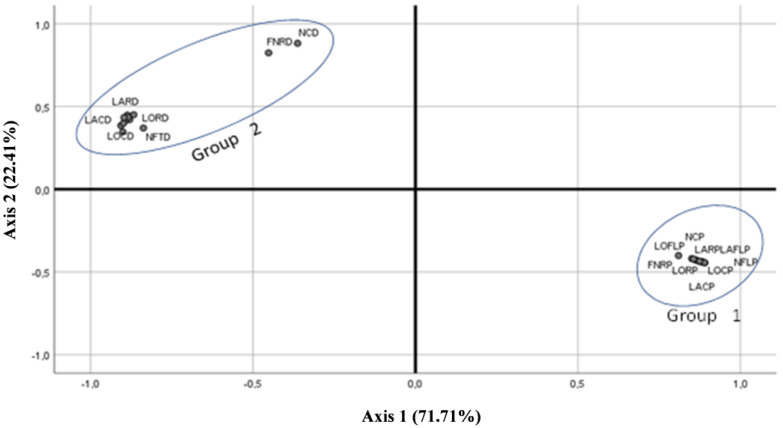
Projection of the quantitative characteristics of the two varieties studied on the first two principal axes of the PCA. LORP: Root length of *A.P* var. *pyrethrum*; LARP: Root Width of *A.P* var. *pyrethrum*; FNRP: Number of branches/individual of *A.P* var. *pyrethrum*; NCP: Number of capitula/individual of *A.P* var. *pyrethrum*; LOCP: capitula length of *A.P* var. *pyrethrum*; LACP: capitula Width of *A.P* var. *pyrethrum*; NFLP: Ligulate flowers Number/capitula of *A.P* var. *pyrethrum*; LAFLP: Ligulate flowers Width of *A.P* var. *pyrethrum*; LOFLP: Ligulate flowers length of *A.P* var. *pyrethrum*; NFTP: Tubular flowers Number/capitula of *A.P* var. *pyrethrum*; LOFTP: Tubular flowers length of *A.P* var. *pyrethrum*; LAFTP: Tubular flowers Width of *A.P* var. *pyrethrum*; LORD: root length of *A.P* var. *depressus*; LARD: Root Width of *A.P* var. *depressus*; FNRD: Number of branches/individual of *A.P* var. *depressus*; NCD: Number of capitula/individual of *A.P* var. *depressus*; LOCD: capitula length of *A.P* var. *depressus*; LACD: capitula Width of *A.P* var. *depressus*; NFLD: Ligulate flowers Number/capitula of *A.P* var. *depressus*; LAFLD: Ligulate flowers Width of *A.P* var. *depreessus*; LOFLD: Ligulate flowers length of *A.P* var. *depressus*; NFTD: Tubular flowers Number/capitula of *A.P* var. *depressus*; LOFTD: Tubular flowers length of *A.P* var. *depressus*; LAFTD: Tubular flowers Width of *A.P* var. *depressus*.

In our PCA analysis, the first principal component explains 71.71% of variability and the second 22.41%. This gives us a cumulative variability of 94.12%. The high representativeness of axis 1 indicates a strong morphological organisation of the two varieties studied. The projection of the quantitative characteristics onto the plane defined by axes 1 and 2 (Figure 2) shows the formation of two groups of characteristics. Group 1, located on the positive side of axis 1, consists of the characters relating to the variety *A.P* var. *pyrethrum*, whereas the second group contains the characters relating to the variety *A.P* var. *depressus*. This subdivision shows that the grouped characters probably represent two morphologically different taxa, at least for the characteristics studied.

The results are similar using either qualitative or quantitative characterisation, as can be seen by the similarity in Figure 1 and Figure 2.

Correlations between the quantitative characteristics show a strong relationship between the characteristics describing the same variety, whether it is *A.P* var. *pyrethrum* or *A.P* var. *depressus.* The dimensions of the floral parts (capitula, ligulate flowers, and tubular flowers) are strongly related to each other; the wider the capitula, the longer it will be, and the lower the number of ligulate and tubular flowers, the wider and longer these flowers will be. A significant positive correlation was observed between the number of branches and the number of capitula per plant, which can be considered an indicator of fruit yield per plant.

These results are in agreement with those of other studies [78,79,80], which showed a positive correlation between plant height, number of branches, number of fruits per plant, and leaf length and width. Factorial correspondence analysis indicates that the five most discriminating qualitative characters are petal back colour, root colour, wing shape, seed colour, and leaf base aspect.

Analysis of qualitative and quantitative morphological characteristics shows that there is a difference between the two varieties studied, which is in line with previous work by Humphries and Ouarghidi [26,28], who showed morphological differences in leaves, flowers, roots, and seeds between the two varieties.

The evaluation of the two varieties for quantitative and qualitative morphological characters of the flowers, roots, seeds, or leaves is a good means for the differentiation of the taxa. In fact, the whole set of examined characters allows us to separate the studied varieties into two different taxa.

### 2.2. Phytochemical Characterisation

#### 2.2.1. Phytochemical Screening

Results of the phytochemical screening carried out on the hydroalcoholic extracts of the different parts of *A.P* var. *pyrethrum* and *A.P* var. *depressus* are shown in Table 5.

The results of the phytochemical screening tests of the different parts of *A.P* var. *pyrethrum* and *A.P* var. *depressus* shown in Table 5 indicate the presence of several chemical compounds. Tannins are present in all parts except in the empty capitula and leaves of *A.P* var. *pyrethrum* (CPP, FPP) and in the seeds of both varieties (GPP, GPD). Flavonoids are present in all extracts, with a high concentration in the roots of *A.P* var. *depressus* (RPD). Sterols and terpenes are detected in the two varieties, with higher concentrations in roots and seeds than in empty capitula, while they are absent in leaves. Alkaloids are present in all parts of the two varieties, but in small amounts in the roots and seeds compared to the leaves and empty capitula. The moss indices show that the content of saponins is high in the empty capitula of the variety *A.P* var. *depressus* (CPD), while they are clearly absent in the leaves of the two varieties (FPP and FPD) and the roots of the variety *A.P* var. *pyrethrum* (RPP). Free quinone is present in *A.P* var. *pyrethrum* (GPP) seeds, while it is absent in *A.P* var. *depressus* (GPD) seeds. Cardiac glycosides, oses, and holosides are absent in the seeds and roots of the two varieties. Finally, it should be noted that mucilage is absent in all the extracts studied. The phytochemical characterisation of the two studied varieties is essential to identifying bioactive molecules. Some of these results are consistent with previous work by [50,63,81,82,83], which showed the presence of flavonoids, alkaloids, and tannins, as well as the presence of mucilage in methanolic extracts of *Anacyclus pyrethrum* (L.). However, hydroethanolic extracts reveal the absence of mucilage. Our phytochemical tests carried out for the first time on the different parts of the two varieties, *A.P* var. *depressus* and *A.P* var. *pyrethrum*, demonstrated the presence of alkaloids, tannins, sterols, and triterpenes, as well as oses and holosides, in the seeds, leaves, empty capitula, and roots of the two varieties.

Through phytochemical screening, we were able to identify and characterise the chemical composition of different parts of the two studied varieties. The test revealed a difference in the content and profile of compounds between the two varieties, which may explain the differences observed in their biological activities [49,58,84].

#### 2.2.2. Physicochemical Characterisation by UHPLC

The different extracts were analysed by ultra-high-performance liquid chromatography at the Institute of Polymers, Composites and Biomaterials (IPCB-CNR), Italy. The details of the main compounds are presented in Table 6.

The chemical composition analysis of the two varieties’ extracts shows a quantitative and qualitative variation in the chemical profile, depending on the part and variety studied. The results of the chromatographic analyses show that caffeic acid, geraniol, and deca-2E,4E-dienoic acid N-Me IBA were detected only in the variety *A.P* var. *pyrethrum*, with the presence of caffeic acid just in the empty capitula, and deca-2*E*,4*E*-dienoic acid N-Me IBA only in the seeds. On the other hand, catechin, chlorogenic acid, p-cumaric acid, ferulic acid, trans-ferulic acid, hesperetin, quercetin, and ((2*E*,4*E*)-*N*-(2-methylpropyl)tetradeca-2,4-diene-8,10-diynamide) were limited to the variety *A.P* var. *depressus*, with the presence of catechin, chlorogenic acid, and p-cumaric acid only in the roots, while myricetin and ferulic acid and ((2*E*,4*E*)-*N*-(2-methylpropyl)tetradeca-2,4-diene-8,10-diynamide) were detected only in the leaves, and trans-ferulic acid in the seeds. However, examination of the extracts revealed the presence of pellitorine, coumarin, and L-arginine in all parts of the two varieties. According to the results of the high-performance liquid chromatography analysis of the extracts, 21 compounds were detected: 16 are new compounds that have never been reported in *A. pyrethrum*, such as caffeic acid, hydroxytyrosol, L-arginine, catechin, vanillic acid, chlorogenic acid, coumarin, cinnamic acid, P-coumaric acid, oleuropein, naringin, quercetin, geraniol, hesperetin, transferulic acid, and ferulic acid. The difference between our analysis and previous analyses could depend on several factors: the location and season of plant collection may result in a change in the active components, and the type of extraction solvent may change the active compounds extracted from the plant samples [71]. The bibliography reports the presence of mainly alkamides [69,77,85,86], principally based on isobutylamide, the main ones being pellitorine and anacycline [37,39,40,41,42,70,81,87,88], which have a wide range of biological acivities, such as antimicrobial, antiviral, diuretic, antioxidant, and analgesic [89,90,91,92], including pellitorine, the main constituent, isolated in 1895 by Dunstan and Garnett [85,93]. Other studies have demonstrated that the plant roots contain hydrocaroline, inulin, sesamin, palmitic acid, hexadecenoic acid, octadecanoic acid, eugenol, and also traces of volatile oil [31,32,42,71,72,73,74,76,77]. Almost all the identified components have been studied for their pharmacological effects, such as gallic acid, known for its anti-tumoral, pro-apoptotic, anti-inflammatory, and antioxidant properties [94,95,96,97,98,99]. Caffeic acid has been shown to have antibacterial, antiviral, antioxidant, anti-inflammatory, anti-atherosclerotic, immunostimulant, antidiabetic, cardioprotective, antiproliferative, hepatoprotective, anticancer, and hepatocellular carcinoma activities [100,101,102,103,104,105,106]. Ferulic acid has been reported to have numerous therapeutic effects, including antioxidant, antimicrobial, anti-inflammatory, antithrombotic, and anticancer activities [107,108]. Chlorogenic acid has been reported to have antioxidant, anti-inflammatory, analgesic, antipyretic, antiviral, anticancer activities [109,110,111,112,113,114]. Catechin has anti-obesity, anti-diabetic, cardiovascular, anti-infectious, hepatoprotective, and neuroprotective properties [115]. Quercetin has great therapeutic potential in the prevention and treatment of various cardiovascular and neurodegenerative diseases, as well as cancer [116,117,118,119,120]. Hesperetin has been reported to have antioxidant, anticarcinogenic, antidiabetic, and many other properties [93,121,122,123]. Furthermore, the results obtained revealed a phytochemical difference between the two varieties *A.P* var. *pyrethrum* and *A.P* var. *depressus.* The richness of the studied extracts in these bioactive compounds could justify the therapeutic use of the different parts of the two varieties [49,58].

### 2.3. Genetic Characterisation

**Figure 3:**
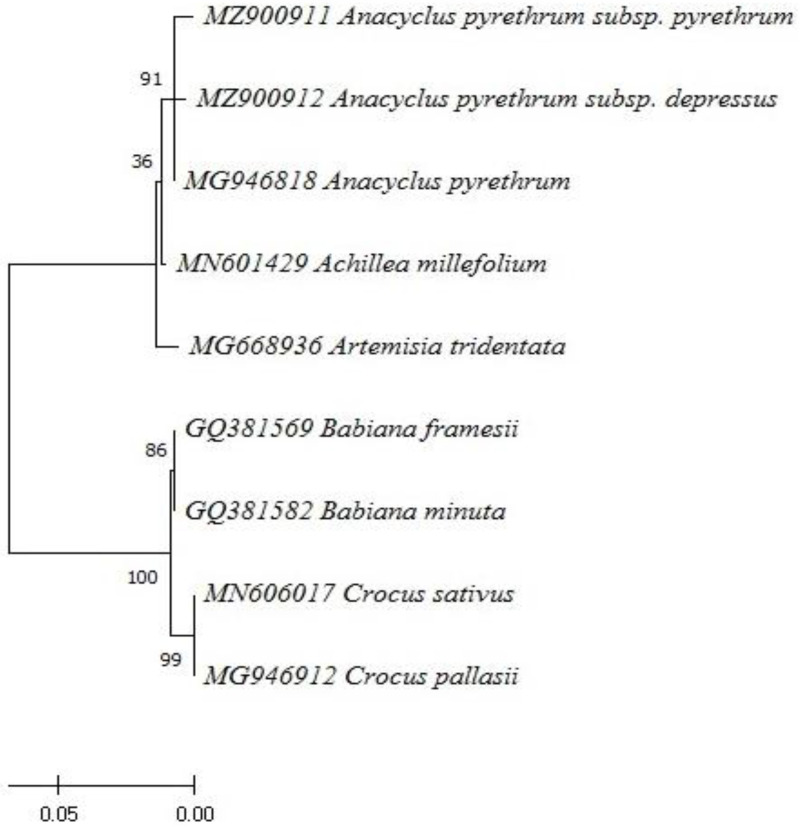
Phylogenetic tree of the two varieties *Anacyclus pyrethrum* var. *pyrethrum* (MZ900911) and *Anacyclus pyrethrum* var. *depressus* (MZ900912) constructed using the maximum likelihood method based on the rbcL (Ribulose-1,5-Bisphosphate Carboxylase) gene.

The amplification of the rbcL (Ribulose-1,5-Bisphosphate Carboxylase) gene in the two samples tested showed a PCR product of ±500 bp. Samples D1 (*A.P* var. *pyrethrum*) and D4 (*A.P* var. *depressus*) are well amplified. Blast analysis using the NCBI genebank revealed that the D1 and D4 sequences were 99% similar to the *Anacyclus pyrethrum* (L.) sequence in the genebank. The two sequences were submitted to the GenBank adapted reference database under accession numbers MZ900911 and MZ900912 and were identified as *Anacyclus pyrethrum* var. *pyrethrum* (L.) Link and *Anacyclus pyrethrum* var. *depressus* (Ball) Maire, respectively. Phylogenetic analysis of our sequences was carried out by comparing them to GenBank references using the Maximum Neighbour Join (MNJ) method and the tree was evaluated by bootstrap analysis based on 1000 replicates. Both sequences were classified in a single clade with *Anacyclus pyrethrum* (L.) Link (Figure 3). These results indicate that there is genetic diversity between the two sequences or varieties analysed, with a similarity percentage of 91%.

The similarity between some varieties could be explained by the presence of several physiological and morphological criteria in common, as well as by the history, origin, and ancestry of these varieties. However, related varieties are classified together [124]. Several studies have been carried out to analyse the genetic diversity of the genus *Anacyclus* [125,126,127,128], the results of which confirm the relationships distinguished by the genetic analysis between the different species and varieties of the genus. The present study adds to the published data set information on the genetic diversity of the two varieties *A.P* var. *pyrethrum* and *A.P* var. *depressus*. However, a full molecular study is needed to provide stronger evidence to elevate *A.P* var. *pyrethrum* and *A.P* var. *depressus* to subspecies status.

## 3. Materials and Methods

### 3.1. Plant Material

*A.P* var. *depressus* and *A.P* var. *pyrethrum* were collected from the Timahdite regions (Tassemakt al maadane). The botanical identification was done with the determination keys (the practical flora of Morocco, volume 3, and the New Flora of Algeria and the Southern Desert Regions) [129,130]. The specimens were kept at the Laboratory of Biotechnology, Environment, Agri-food and Health (LBEAS), Faculty of Sciences Dhar el mahraz Fez, Morocco (specimen voucher n° A31/31-5-18/TM; A32/31-5-18/TM).

### 3.2. Morphological Characterisation

In order to carry out a complete morphological characterisation of the two varieties, a list of descriptors was first established from the observation of individuals of each variety. Then, only those descriptors that could be determined with the available equipment (ruler, meter, calliper, and binocular magnifier) and in a fairly objective manner were selected. 25 plants per variety, selected at random, were assessed for morphological traits related to vegetative and floral development. The morphological characterisation of the two varieties was established on the basis of 23 characteristics: 16 quantitative and 7 qualitative characteristics, describing the vegetative and floral parts, were selected. All measurements and descriptions were made on the leaves, flowers, capitulas, seeds, and roots of each variety. The width and length were measured with a 30 cm ruler. The colours of the different parts of the flowers, seeds, and roots were assigned using the Royal Horticulture Society colour chart. Phenotyping of the vegetative and floral parts was carried out between April and July.

The variability of quantitative characteristics within each variety was determined by calculating the coefficient of variation (CV) of each characteristic according to the following formula:

CV = standard deviation/mean of the data set

### 3.3. Phytochemical Characterisation

#### 3.3.1. Preparation of Extracts

The different parts (leaves, empty capitulas, seeds, and roots) of the two varieties, *A.P* var. *depressus* and *A.P* var. *pyrethrum*, were harvested and air-dried for a fortnight, then pulverised with an electric grinder and kept in the laboratory until the day of extraction. Extracts were prepared by cold maceration of 50 g of powder of different parts (roots, seeds, leaves, and empty capitulas) of the two varieties studied in 500 mL of 70% ethanol, for 48 h in the dark at room temperature. The macerates were filtered through Whatman paper. The solvent was removed by vacuum evaporation at a moderate temperature (40 °C), and the residue obtained was then stored at 4 °C until further use. The ethanolic extract was chosen based on its strong ability to extract a wide range of active compounds, its fast execution, easy evaporation, and lower harm to humans and the environment (green solvent).

#### 3.3.2. Phytochemical Screening

In order to verify the presence or absence of some phytochemical compounds that can be present in plant extracts, we have performed some classical tests based on colorimetric reactions and precipitation by specific chemical reagents [63,131,132,133,134,135,136,137,138,139]. The results are classified according to their appearance as follows:

- Frankly positive reaction: +++;
- Positive reaction: ++;
- Moderately positive reaction: +;
- Negative reaction: −.

#### 3.3.3. Physicochemical Characterisation by UHPLC

The extracts were analysed using a Shimadzu Ultra-High-Performance Liquid Chromatography system (Nexera XR LC 40) coupled to an MS/MS detector (LCMS 8060, Shimadzu Italy, Milan, Italy). The MS/MS operated with electrospray ionisation (ESI) and was controlled by Lab Solution software, allowing for quick switching between low energy scan (4V, full scan MS) and high energy scan (10–60 V ramping) during a single LC run. The source parameters were set as follows: nebulising gas flow of 2.9 L/min, heating gas flow of 10 L/min, interface temperature of 300 °C, DL temperature of 250 °C, heat block temperature of 400 °C, and drying gas flow of 10 L/min. The analysis was conducted using flow injection with the mobile phase composed of acetonitrile/water + 0.01% formic acid (5:95, *v*/*v*). The instrument was configured for a selected ion monitoring (SIM) experiment in negative mode, with only syringic acid detected in positive ESI. Compound identification was performed by comparison with retention times of database compounds and confirmed by their characteristic fragmentations obtained in flow injection with a mobile phase consisting of acetonitrile: water + 0.01% formic acid (5:95, *v*/*v*).

### 3.4. Molecular Characterisation

#### 3.4.1. DNA Extraction

In a first step, 3–5 leaves of similar age per variety were randomly sampled. DNA extraction was done according to the protocol described by Cota-Sánchez et al. [140]. In short, plant samples are prepared by cryogenic grinding of tissues after cooling in liquid nitrogen. Mix 100 mg of homogenised tissue with 500 µL of CTAB extraction buffer and vortex carefully, then transfer the homogenate to a 60 °C bath for 30 min. After the incubation period, centrifuge the homogenate for 5 min at 14,000× *g*, then transfer the supernatant to a new tube, add 5 µL of RNase A solution, and incubate at 37 °C for 20 min. Add an equal volume of chloroform/isoamyl alcohol (24:1), vortex for 5 s, then centrifuge the sample for 1 min at 14,000× *g* to separate the phases, transfer the upper aqueous phase to a new tube, and repeat this extraction until the upper phase is clear. Then, transfer the upper aqueous phase to a new tube, precipitate the DNA by adding 0.7 volume of cold isopropanol, and incubate at −20 °C for 15 min. Centrifuge the sample at 14,000× *g* for 10 min, decant the supernatant without disturbing the pellet, wash with 500 µL of ice-cold 70% ethanol, decant the ethanol, remove the residual ethanol by drying in a Speed Vac, dry the pellet long enough to remove the alcohol, and dissolve the DNA in 20 µL of TE buffer (10 mm Tris, ph 8, 1 mm EDTA).

#### 3.4.2. DNA Amplification and Sequencing

The extracted DNA was subjected to PCR using two universal primer sets: rbcL a-f/rbcL a-R and the second set, rpocL1-2/rpocL1-4 (Table 7). PCR reactions were conducted by taking a 25 μL volume that contains 2.5 μL of DNA, 2.5 μL (10×) of PCR buffer, 0.25 μL (10 mM of each) dNTP, 2 μL (50 mM) of MgCl_2_, 1 μL (10 µM) of primers, and 0.5 μL (5 µ/µL) of Taq DNA polymerase [141]. The remaining volume is made up with sterile distilled water. The amplifications were performed following the conditions described in Table 8 for each primer. PCR products were then examined using a 1% electrophoresis gel. Sequencing analysis was performed using the Sanger method (Table 8). Sequences were then processed and aligned using BioEdit software (version 7.0.5.3), and similarity was checked in Genbank prior to classification using the Blast program.

Rbcl: ribosomal protein; Rpoc: RNA polymerase beta’ subunit.

### 3.5. Statistical Analysis

Descriptive statistics were calculated using Microsoft Office Excel 2016. Anova was used to study the intra- and interpopulation variations of the two varieties, and GraphPad Prism 7.0 was used for the analyses. In order to confirm the relationships between the quantitative traits and to determine the most discriminating qualitative traits, principal component analysis (PCA), and correspondence factor analysis (CFA) were performed.

## 4. Conclusions

The present study revealed that, based on the morphological variation of the two varieties studied, phytochemical and genetic variations were observed. At the same time, the chromatographic analysis of the extracts showed a variation in the chemical profile depending on the part and variety studied, as well as the presence of compounds that have never been reported in *A. pyrethrum*, many of them with recognized health promoting effects. 

## Figures and Tables

**Table 1:** Qualitative morphological descriptors analysed for the two varieties.

Qualitative Characteristics	*A.P* var. *pyrethrum*	*A.P* var. *despressus*
Roots		
Colour	Dark brown (CRBFP)	Light brown (CRBCD)
Leaves		
Colour	Glaucous (CFGP)	Glaucous (CFGD)
Base appearance	Evergreen (BFPP)	Not evergreen (BFNPD)
Capitula		
Flower ray colour	Yellow (CRFJP)	Yellow (CRFJD)
Petal back colour	Red (CDPRP)	Violet (CDPVD)
Seeds		
Colour	Dark (CGSP)	Clear (CGCD)
Wing	Thin (GAMP)	Thick (GAED)

CRBFP: dark brown root colour of *A.P* var. *pyrethrum*; CGSP: dark seed colour of *A.P* var. *pyrethrum*; CDPRP: red petal back colour of *A.P* var. *pyrethrum*; BFPP: evergreen leaf base of *A.P* var. *pyrethrum*; GAMP: thin wings of *A.P* var. *pyrethrum*; CFG: glaucous leaves; CRFJ: yellow floral ray; CDPVD: violet Petal back colour of *A.P* var. *depressus*; CGCD: clear seed colour of *A.P* var. *depressus*; BFNPD: not evergreen base of *A.P* var. *depressus*; GAED: thick wings of *A.P* var. *depressus*; CRBCD: light brown root colour of *A.P* var. *depressus*.

**Table 2:** Quantitative morphological descriptors analysed for *A.P* var. *depressus*.

Variables	Minimum Value	Maximum Value	Mean/Standard Deviation
Roots			
Length (cm) (LOR)	5	9	6.637 ± 1.110
Width (cm) (LAR)	0.9	1.3	1.065 ± 0.142
Leaves			
Number of branches/individual (FNR)	41	102	52.38 ± 20.188
Capitula			
Number/individual (NC)	50	320	89.32 ± 29.80
Length (cm) (LOC)	0.7	1.2	0.958 ± 0.139
Width (cm) (LAC)	0.8	1.2	0.97 ± 0.138
Ligulate flowers			
Number/capitula (NFL)	12	15	13.15 ± 0.978
Length (mm) (LOFL)	7.8	13	9 ± 0.105
Width (mm) (LAFL)	2	3	2.4 ± 0.038
Tubular flowers			
Number/capitula (NFT)	34	130	78.05 ± 25.920
Length (mm) (LOFT)	3	5.6	4.21 ± 0.090
Width (mm) (LAFT)	1	1.2	1.02 ± 0.006
Seeds			
Number/capitula (NG)	40	143	81.73 ± 22.45
Length (mm) (LOG)	2.8	3.5	3.267 ± 0.404
Width (mm) (LAG)	2.2	2.6	2.433 ± 0.208
Weight of 100 seeds (g) (PG)	0.04	0.06	0.05 ± 0.005

**Table 3:** Quantitative morphological descriptors analysed for *A.P* var. *pyrethrum*.

Variables	Minimum Value	Maximum Value	Mean/Standard Deviation
Roots			
Length (cm) (LOR)	10	18	13.979 ± 2.188
Width (cm) (LAR)	0.9	1.8	1.424 ± 0.282
Leaves			
Number of branches/individual (FNR)	16	63	34.15 ± 10.80
Capitula			
Number/individual (NC)	29	69	46.33 ± 10.094
Length (cm) (LOC)	1.3	2.3	1.79 ± 0.247
Width (cm) (LAC)	1.3	2.2	1.714 ± 0.224
Ligulate flowers			
Number/capitula (NFL)	9	13	10.92 ± 1.284
Length (mm) (LOFL)	14	17	15.44 ± 1.031
Width (mm) (LAFL)	2.1	4.2	3.192 ± 0.79
Tubular flowers			
Number/capitula (NFT)	36	188	117.36 ± 27.509
Length (mm) (LOFT)	5.9	8	7.16 ± 0.56
Width (mm) (LAFT)	1.2	2.6	1.913 ± 0.30
Seeds			
Number/capitula (NG)	81	175	116.98 ± 21.75
Length (mm) (LOG)	3.9	4.2	4.033 ± 0.153
Width (mm) (LAG)	3.6	4	3.767 ± 0.208
Weight of 100 seeds (g) (PG)	0.11	0.14	0.13 ± 0.01

**Table 4:** Descriptive statistics for quantitative morphological characteristics.

Variables	Variation Coefficient between Individuals of *A.P* var. *depressus*	Variation Coefficient between Individuals of *A.P* var. *pyrethrum*	Variation Coefficient between the Two Varieties	Significance *p* = 0.001
Roots				
Length (cm) (LOR)	16.72%	15.65%	39.45%	***
Width (cm) (LAR)	13.37%	19.81%	23.02%	ns
Leaves				
Number of branches/individual (FNR)	24.86%	31.62%	34.71%	***
Capitula				
Number/individual (NC)	33.36%	21.78%	45.47%	**
Length (cm) (LOC)	14.55%	13.83%	33.67%	ns
Width (cm) (LAC)	14.31%	13.11%	31.06%	ns
Ligulate flowers				
Number/capitula (NFL)	7.43%	11.76%	13.25%	ns
Length (mm) (LOFL)	16.05%	6.67%	48.83%	***
Width (mm) (LAFL)	11.72%	24.96%	26.35%	*
Tubular flowers				
Number/capitula (NFT)	33.21%	23.44%	33.92%	ns
Length (mm) (LOFT)	21.50%	7.93%	29.11%	*
Width (mm) (LAFT)	5.94%	16.08%	33.83%	ns
Seeds				
Number/capitula (NG)	25.59%	18 %	29.50%	***
Length (mm) (LOG)	12.36%	3.79%	14.35%	ns
Width (mm) (LAG)	8.54%	5.52%	23.40%	ns
Weight of 100 seeds (g) (PG)	10%	7.69%	43.85%	**

* *p* < 0.05; ** *p* < 0.01; *** *p* < 0.001; ns: not significant.

**Table 5:** Phytochemical screening of the hydroethanol extracts of the different parts of the two varieties *A.P* var. *pyrethrum* and *A.P* var. *depressus*.

Compounds/Extracts	*A.P* var. *pyrethrum*				*A.P* var. *depressus*			
	Capitula (CPP)	Seeds (GPP)	Roots (RPP)	Leaves (FPP)	Capitula (CPD)	Seeds (GPD)	Roots (RPD)	Leaves (FPD)
Tannins	−	−	+	−	+	−	+	+
Catechic tannins	−	−	+	−	−	−	+	+
Gallic tannins	−	−	−	−	+	−	−	−
Flavonoids	+	++	+	++	++	++	+++	++
Sterols	+	+	−	+	+	+	+	+
Alkaloids								
Dragondorf test	+	++	++	+	+	+	++	+
Mayer’s test	++	+	++	++	++	+	+	++
Saponosides	+	+	−	−	+++	+	+	−
Cardiac glycosides	+	−	−	+	+	−	−	+
Oses and holosides	+	−	−	+	+	−	−	+
Mucilages	−	−	−	−	−	−	−	−
Free quinones	+	+++	−	++	+	−	−	+++
Sterols and terpenes	+	+++	+++	−	++	+++	+++	−
Steroidal heterosides	++	+	−	++	++	+	−	++
Triterpenes heterosides	+	+	+	++	+	+	+	++

Strongly positive reaction (+++); positive reaction (++); moderately positive reaction (+); negative reaction (−).

**Table 6:** Chemical composition obtained by UHPLC of the different parts (roots, seeds, leaves, and capitula) of the two varieties *A.P* var. *pyrethrum* and *A.P* var. *depressus*.

No	RT	*m*/*z*	Structural Formula	Compounds	% Area							
					*A.P* var. *pyrethrum*				*A.P* var. *depressus*			
					Roots (RPP)	Seeds (GPP)	Leaves (FPP)	Capitula (CPP)	Roots (RPD)	Seeds (GPD)	Leaves (FPD)	Capitula (CPD)
**1**	0.60	180	C_9_H_8_O_4_	Caffeic acid	−	−	−	0.55	−	−	−	−
**2**	4.60	154	C_8_H_10_O_3_	Hydroxytyrosol	1.11	5.56	2.62	3.02	1.37	3.73	1.89	3.87
**3**	5.95	174	C_6_H_14_N_4_O_2_	L-arginine	15.76	16.12	2.29	3.20	15.02	1.68	1.49	2.82
**4**	6.64	170	C_7_H_6_O_5_	Gallic acid	6.52	6.09	8.95	11.00	−	4.54	7.16	11.91
**5**	7.26	223	C_14_H_25_NO	Pellitorine	0.18	1.07	0.85	1.25	0.15	0.35	0.57	0.46
**6**	12.77	290	C_15_H_14_O_6_	Catechin	−	−	−	−	7.23	−	−	−
**7**	16.98	168	C_8_H_8_O_4_	Vanillic acid	3.07	−	1.10	4.60	0.45	−	1.11	2.28
**8**	17.61	354	C_16_H_18_O_9_	Chlorogenic acid	−	−	−	−	1.05	−	−	−
**9**	21.69	146	C_9_H_6_O_2_	Coumarin	2.40	12.56	19.29	12.98	3.37	11.41	20.53	13.73
**10**	21.79	148	C_9_H_8_O_2_	Cinnamic acid	1.07	−	−	−	2.42	−	−	−
**11**	21.86	164	C_9_H_8_O_3_	P-coumaric acid	−	−	−	−	0.88	−	−	−
**12**	22.03	194	C_10_H_10_O_4_	Transferulic acid	−	−	−	−	−	1.00	−	−
**13**	22.15	194	C_10_H_10_O_4_	Ferulic acid	−	−	−	−	−	−	1.52	−
**14**	23.21	540	C_25_H_32_O_13_	Oleuropein	−	0.41	3.07	0.84	−	0.32	1.65	0.44
**15**	24.63	580	C_27_H_32_O_14_	Naringin	−	0.58	0.46	0.44	−	0.31	0.26	−
**16**	24.86	302	C_15_H_10_O_7_	Quercetin	−	−	−	−	−	−	0.18	−
**17**	25.96	154	C_10_H_18_O	Geraniol	−	−	3.67	1.49	−	−	−	−
**18**	28.59	302	C_16_H_14_O_6_	Hesperetin	−	−	−	−	6.79	2.83	8.36	
**19**	38.72	237	C_15_H_27_NO	Deca-2*E*,4*E*-dienoic acid N-Me IBA	−	0.39	−	−	−	−	−	−
**20**	42.03	271	C_18_H_25_NO	Anacyclin	0.67	−	−	−	0.44	−	−	−
**21**	54.25	318	C_15_H_10_O_8_	((2*E*,4*E*)-*N*-(2-methylpropyl)tetradeca-2,4-diene-8,10-diynamide)	−	−	−	−	−	−	0.27	−

**Table 7:** Characteristics of the four marker primers used for amplification.

Primer Name	Sequence	Number of Nucleotides
***rbcL a-f***	5′ ATG TCA CCA CAA ACA GAG ACT AAA GC3′	26
***rbcL a-r***	5′ GTA AAA TCA AGT CCA CCG CG 3′	20
***rpoC1-2***	5′ GGC AAA GAG GGA AGA TTT CG3′	20
***rpoC1-4***	5′ CCA TAA GCA TAT CTT GAG TTG G 3′	22

**Table 8:** PCR reaction conditions.

Reaction Condition	Locus	
	*rbcL*	*rpoC1*
**Initial denaturation**	95 °C—4 min	94 °C—1 min
**Denaturation**	94 °C—30 s	94 °C—30 s
**Annealing**	55 °C—1 min	50 °C—40 s
**Extension**	72 °C—1 min	72 °C—40 s
**Final extension**	72 °C—10 min	72 °C—5 min
**Number of cycles**	35	40

## Data Availability

Not applicable.

## Supplementary Materials

The following supporting information can be downloaded at: https://www.mdpi.com/article/10.3390/molecules28145378/s1, Table S1: Matrix of correlation coefficients between the different variables measured.

## Sample Availability

The data from this work are available in the manuscript as well as in the electronic Supplementary Materials File.
