# Supplementary material for: Phytochemical, Morphological and Genetic Characterisation of Anacyclus pyrethrum var. depressus (Ball.) Maire and Anacyclus pyrethrum var. pyrethrum (L.) Link
Source: Molecules. 2023 Jul 13;28(14):5378. doi: 10.3390/molecules28145378 (PMC10385216; doi:10.3390/molecules28145378)
Supplement: Supplementary file 1 [file molecules-28-05378-s001.zip › molecules-2412758-supplementary.pdf]

Table S1. Matrix of correlation coefficients between the different variables measured.

	LORP	LARP	FNRP	NCP	LOCP	LACP	NFLP	LAFLP	LOFLP	NFTP	LOFTP	LAFTP	LORD	LARD	FNRD	NCD	LOCD	LACD	NFLD	LAFLD	LOFLD	NFTD	LOFTD	LAFTD
LORP	1.000	0.942	0.876	0.926	0.960	0.962	0.965	0.972	0.925	0.919	0.968	0.949	-0.951	-0.960	-0.755	-0.699	-0.957	-0.957	-0.971	-0.953	-0.964	-0.885	-0.935	-0.973
LARP	0.942	1.000	0.867	0,922	0,940	0,941	0,948	0,960	0,914	0,922	0,959	0,942	-0,938	-0,947	-0,745	-0,690	-0,944	-0,944	-0,958	-0,940	-0,950	-0,873	-0,922	-0,960
FNRP	0.876	0.867	1.000	0,859	0,874	0,878	0,872	0,895	0,841	0,859	0,881	0,856	-0,877	-0,886	-0,697	-0,645	-0,883	-0,884	-0,896	-0,879	-0,889	-0,817	-0,863	-0,898
NCP	0.926	0.922	0.859	1,000	0,939	0,941	0,945	0,958	0,920	0,914	0,951	0,928	-0,931	-0,940	-0,739	-0,684	-0,937	-0,937	-0,951	-0,933	-0,943	-0,866	-0,915	-0,953
LOCP	0.960	0.940	0.874	0,939	1,000	0,998	0,978	0,975	0,923	0,934	0,979	0,966	-0,955	-0,965	-0,759	-0,703	-0,962	-0,962	-0,976	-0,957	-0,968	-0,889	-0,940	-0,978
LACP	0.962	0.941	0.878	0,941	0,998	1,000	0,980	0,977	0,925	0,933	0,980	0,965	-0,957	-0,966	-0,760	-0,704	-0,963	-0,964	-0,978	-0,959	-0,970	-0,891	-0,941	-0,980
NFLP	0.965	0.948	0.872	0,945	0,978	0,980	1,000	0,978	0,925	0,942	0,982	0,962	-0,960	-0,970	-0,763	-0,706	-0,967	-0,967	-0,981	-0,962	-0,973	-0,894	-0,944	-0,983
LAFLP	0.972	0.960	0.895	0,958	0,975	0,977	0,978	1,000	0,960	0,947	0,989	0,971	-0,969	-0,978	-0,770	-0,713	-0,975	-0,976	-0,990	-0,971	-0,982	-0,902	-0,953	-0,992
LOFLP	0.925	0.914	0.841	0,920	0,923	0,925	0,925	0,960	1,000	0,899	0,938	0,916	-0,918	-0,927	-0,729	-0,675	-0,924	-0,925	-0,938	-0,920	-0,931	-0,855	-0,903	-0,940
NFTP	0.919	0.922	0.859	0,914	0,934	0,933	0,942	0,947	0,899	1,000	0,952	0,935	-0,924	-0,933	-0,734	-0,680	-0,930	-0,931	-0,945	-0,926	-0,937	-0,860	-0,909	-0,946
LOFTP	0.968	0.959	0.881	0,951	0,979	0,980	0,982	0,989	0,938	0,952	1,000	0,989	-0,967	-0,977	-0,768	-0,711	-0,974	-0,974	-0,988	-0,969	-0,981	-0,900	-0,951	-0,990
LAFTP	0.949	0.942	0.856	0,928	0,966	0,965	0,962	0,971	0,916	0,935	0,989	1,000	-0,949	-0,959	-0,754	-0,698	-0,956	-0,956	-0,970	-0,951	-0,962	-0,884	-0,934	-0,972
LORD	-0.951	-0.938	-0.877	-0,931	-0,955	-0,957	-0,960	-0,969	-0,918	-0,924	-0,967	-0,949	1,000	0,962	0,745	0,690	0,954	0,956	0,968	0,949	0,957	0,901	0,931	0,971
LARD	-0.960	-0.947	-0.886	-0,940	-0,965	-0,966	-0,970	-0,978	-0,927	-0,933	-0,977	-0,959	0,962	1,000	0,753	0,682	0,965	0,966	0,974	0,961	0,971	0,902	0,941	0,979
FNRD	-0.755	-0.745	-0.697	-0,739	-0,759	-0,760	-0,763	-0,770	-0,729	-0,734	-0,768	-0,754	0,745	0,753	1,000	0,789	0,720	0,725	0,769	0,760	0,756	0,680	0,688	0,762
NCD	-0.699	-0.690	-0.645	-0,684	-0,703	-0,704	-0,706	-0,713	-0,675	-0,680	-0,711	-0,698	0,690	0,682	0,789	1,000	0,680	0,690	0,716	0,711	0,704	0,640	0,648	0,709
LOCD	-0.957	-0.944	-0.883	-0,937	-0,962	-0,963	-0,967	-0,975	-0,924	-0,930	-0,974	-0,956	0,954	0,965	0,720	0,680	1,000	0,991	0,973	0,954	0,965	0,897	0,966	0,980
LACD	-0.957	-0.944	-0.884	-0,937	-0,962	-0,964	-0,967	-0,976	-0,925	-0,931	-0,974	-0,956	0,956	0,966	0,725	0,690	0,991	1,000	0,972	0,953	0,968	0,887	0,954	0,979
NFLD	-0.971	-0.958	-0.896	-0,951	-0,976	-0,978	-0,981	-0,990	-0,938	-0,945	-0,988	-0,970	0,968	0,974	0,769	0,716	0,973	0,972	1,000	0,974	0,982	0,905	0,950	0,990
LAFLD	-0.953	-0.940	-0.879	-0,933	-0,957	-0,959	-0,962	-0,971	-0,920	-0,926	-0,969	-0,951	0,949	0,961	0,760	0,711	0,954	0,953	0,974	1,000	0,966	0,894	0,930	0,972
LOFLD	-0.964	-0.950	-0.889	-0,943	-0,968	-0,970	-0,973	-0,982	-0,931	-0,937	-0,981	-0,962	0,957	0,971	0,756	0,704	0,965	0,968	0,982	0,966	1,000	0,882	0,943	0,981
NFTD	-0.885	-0.873	-0.817	-0,866	-0,889	-0,891	-0,894	-0,902	-0,855	-0,860	-0,900	-0,884	0,901	0,902	0,680	0,640	0,897	0,887	0,905	0,894	0,882	1,000	0,868	0,906
LOFTD	-0.935	-0.922	-0.863	-0,915	-0,940	-0,941	-0,944	-0,953	-0,903	-0,909	-0,951	-0,934	0,931	0,941	0,688	0,648	0,966	0,954	0,950	0,930	0,943	0,868	1,000	0,967
LAFTD	-0.973	-0.960	-0.898	-0,953	-0,978	-0,980	-0,983	-0,992	-0,940	-0,946	-0,990	-0,972	0,971	0,979	0,762	0,709	0,980	0,979	0,990	0,972	0,981	0,906	0,967	1,000
